# Third-wave cognitive behavioral therapies for caregivers of cancer patients: a scoping review

**DOI:** 10.1186/s12906-023-04186-3

**Published:** 2023-10-11

**Authors:** Bomi Hong, Sora Yang, Sojeong Hyeon, Sojeong Kim, Jiyeon Lee

**Affiliations:** 1 College of Nursing and Brain Korea 21 FOUR Project, Yonsei University, Seoul, South Korea; 2 Graduate School, Yonsei University, Seoul, South Korea; 3 Yonsei Cancer Center, Seoul, South Korea; 4 University-Industry Foundation, Yonsei University, Seoul, South Korea; 5 College of Nursing and Mo-Im Kim Nursing Research Institute, Yonsei University, Seoul, South Korea

**Keywords:** Cancer, Caregiver, Cognitive and behavioral therapy, Mindfulness, Acceptance and commitment therapy

## Abstract

**Background:**

Cancer caregivers extend comprehensive support covering all aspects of patients’ daily lives. It has been reported that a significant proportion of cancer caregivers experience emotional distress. As one way to solve this problem, third-wave cognitive behavioral therapies (CBT), which involves integrating acceptance and mindfulness into cognitive‒behavioral therapy, has been applied to improve caregiver outcomes.

**Methods:**

A scoping review was conducted based on the scoping review guidelines proposed by the Jonna Briggs Institute (JBI). The population was caregivers of cancer patients, the concept was third-wave CBT, and the context remained open. English and Korean publications published from 2001 to June 2022 were identified from PubMed, Embase, CINAHL, PsycINFO, Cochrane, Korea Med, and RISS.

**Results:**

A total of 12 studies were included in this scoping review. Mindfulness-Based Stress Reduction (MBSR) and Acceptance and Commitment Therapy (ACT) was the most frequently applied intervention (n = 3, each). Among the components of third-wave CBT, ‘mindfulness’ was identified in all the studies reviewed (n = 12). Dyadic interventions comprised the majority (n = 9). Interventions using digital technologies such as mobile application/web page (n = 3), telephone (n = 3), and FaceTime (n = 2) have increased since 2017. Depression was the most frequently evaluated outcome (n = 8), followed by anxiety and mindfulness (n = 6, each).

**Conclusions:**

The current review explored available third-wave CBT intervention studies for cancer caregivers and targeted outcomes. Most of the interventions were dyadic interventions and utilized mindfulness. Delivery methods were continuously updated with digital technologies. Further RCTs with robust research designs and a synthesis of the results of the trials would provide evidence about how to effectively apply third-wave CBTs for cancer caregivers.

**Supplementary Information:**

The online version contains supplementary material available at 10.1186/s12906-023-04186-3.

## Background

### Introduction

Caregivers of cancer patients offer extensive support in patients’ daily lives. According to the National Alliance for Caregiving [1], caregivers of cancer patients are more likely to report supporting patients’ activities of daily living in all categories than those caring for elderly individuals, such as caregivers of dementia patients. In terms of caregiving burden, cancer appears to have a more significant impact on the caregiver’s daily schedule, and it has a more substantial financial impact than on caregivers for noncancer patients [2]. Along with the physical and financial implications, many caregivers experience psychological issues. It has been reported that approximately 50% of cancer caregivers experience significant levels of emotional distress, while 37% of noncancer survivors are emotionally distressed [1]. Caregivers of cancer patients are likely to be depressed and anxious and have unmet needs in terms of their emotional well-being [3] and social support [2].

Various psychosocial interventions have been implemented to meet the needs of cancer caregivers. These interventions were intended to provide information and support and enhance caregivers’ coping resources to improve their quality of life and reduce their emotional distress [4]. Among these interventions, third-wave cognitive behavioral therapy (CBT) integrating acceptance and mindfulness into traditional cognitive behavioral therapy provides supportive care for caregivers and therefore warrants further attention.

CBT is an intervention with blended technique of behavioral and cognitive therapy. Behavior therapy focuses on the direct modification of problematic behaviors through operant conditioning and systematic desensitization [5, 6]. Cognitive therapy helps patients gain a rational perspective and implement behavioral change by identifying cognitive distortions and restructuring cognition [5]. Various behavioral strategies have been incorporated with cognitive therapy and have become to be called as cognitive behavioral therapy (CBT). With introduction of the third-wave CBT, it is now called as the second-wave CBT [5].

In the late 1990s and early 2000s, a growing understanding of the limitations of CBT proposed new wave of behavioral therapies. The third-wave CBT emphasizes mindfulness, acceptance, and awareness emerged, aiming to foster a more adaptive and nonjudgmental relationship with human thoughts and feelings [5, 6]. Acceptance and commitment Therapy (ACT), dialectical Behavioral therapy (DBT), and mindfulness-based cognitive therapy (MBCT) are recognized as representative third-wave CBT, and treatments such as cognitive behavioral analysis system of psychotherapy (CBASP), mindfulness-based stress reduction (MBSR), metacognitive therapies (MCT), and integrative behavioral couple therapy (IBCT) could be considered as various forms of the third-wave CBT [7, 8].

Both second- and third-wave CBT are based on behavioral principles and goal-oriented. Whereas the third-wave CBT is intended not to correct dysfunctional beliefs and reduce symptoms but to work toward the empowerment of patients [5, 9]. Third-wave CBT is attracting attention in terms of its use not only in the clinical population but also in the nonclinical population [10, 11].

There are studies that have applied third-wave CBT for caregivers, including acceptance and commitment therapy (ACT) [12], mindfulness-based stress reduction (MBSR) [13], mindfulness-based cognitive therapy (MBCT) [14], and dialectical behavioral therapy (DBT) [13]. Positive effects on anxiety, depression, stress, self-efficacy, mindfulness, and quality of life have been observed in caregivers of diverse patients such as autism, brain damage, cancer and dementia patients [12–14].

However, there is a lack of understanding about trends in third-wave CBT applied to caregivers for cancer patients.

### Objectives

This scoping review aimed to provide an overview of studies that have evaluated the efficacy of third-wave CBT with cancer caregivers.

## Methods

The scoping review was conducted based on the scoping review guidelines proposed by the Jonna Briggs Institute (JBI) [15].

### Identifying the research question

In this review, the population was caregivers of adult cancer patients and survivors, and the concept was third-wave CBT. The context remained open. The detailed research questions are as follows.

● What were the characteristics of cancer caregivers who received third-wave CBT interventions?

● What types of third-wave CBT were applied to and evaluated for caregivers of cancer patients?

● What were the characteristics of third-wave CBT for caregivers of cancer patients, including the delivery methods, duration, and providers?

● What were the targeted outcomes of third-wave CBT, and which measurement instruments were used to measure the target outcome?

### Identifying relevant studies

The search was conducted on June 26, 2022, using six electronic databases, including PubMed, Embase, CINAHL, PsycINFO, Cochrane, Koreamed, and RISS. Search terms included ‘cancer,‘ ‘caregiver,‘ ‘third-wave cognitive behavioral therapy’, and ‘intervention’ (Supplementary Table 1). Search terms for intervention were further specified as acceptance and commitment therapy (ACT), mindfulness-based cognitive therapy (MBCT), mindfulness-based stress reduction (MBSR), behavioral activation (BA), cognitive behavioral analysis system of psychotherapy (CBASP), meta-cognitive therapy (MCT), and dialectical behavior therapy (DBT) based on other reviews on the third-wave CBT [14, 16]. Studies published in English or Korean over the past 20 years were searched, as this reflected the period when third-wave CBT became popular in clinical practice [6, 17].

### Study selection

Through pilot screening, the entire team screened random samples of 25 articles, discussed differences among them, modified the eligibility criteria, and achieved a consensus on more than 75% of the articles. The inclusion criteria were (1) studies on the caregivers of adult cancer patients, (2) studies using third-wave CBT, (3) experimental studies with control groups, (4) studies that measured quantitative outcomes, and (5) studies published in English or Korean for which the full text was available.

A web-based literature review management software, Covidence (Veritas Health Innovation) [18], was used for this scoping review. Two independent researchers reviewed the titles and abstracts to identify studies related to population, concept, and context. Likewise, two investigators independently performed a full-text review and selected the studies that met the criteria.

### Charting the data

The data extraction was conducted utilizing the modified data extraction form from Covidence, which includes the author, country, publication year, research method, general characteristics of participating cancer patients and caregivers, characteristics of the intervention, and variables measured. Two independent researchers performed data extraction on each article, and a third reviewer participated in resolving conflicting results.

## Results

### General characteristics of the included studies

**Fig. 1:**
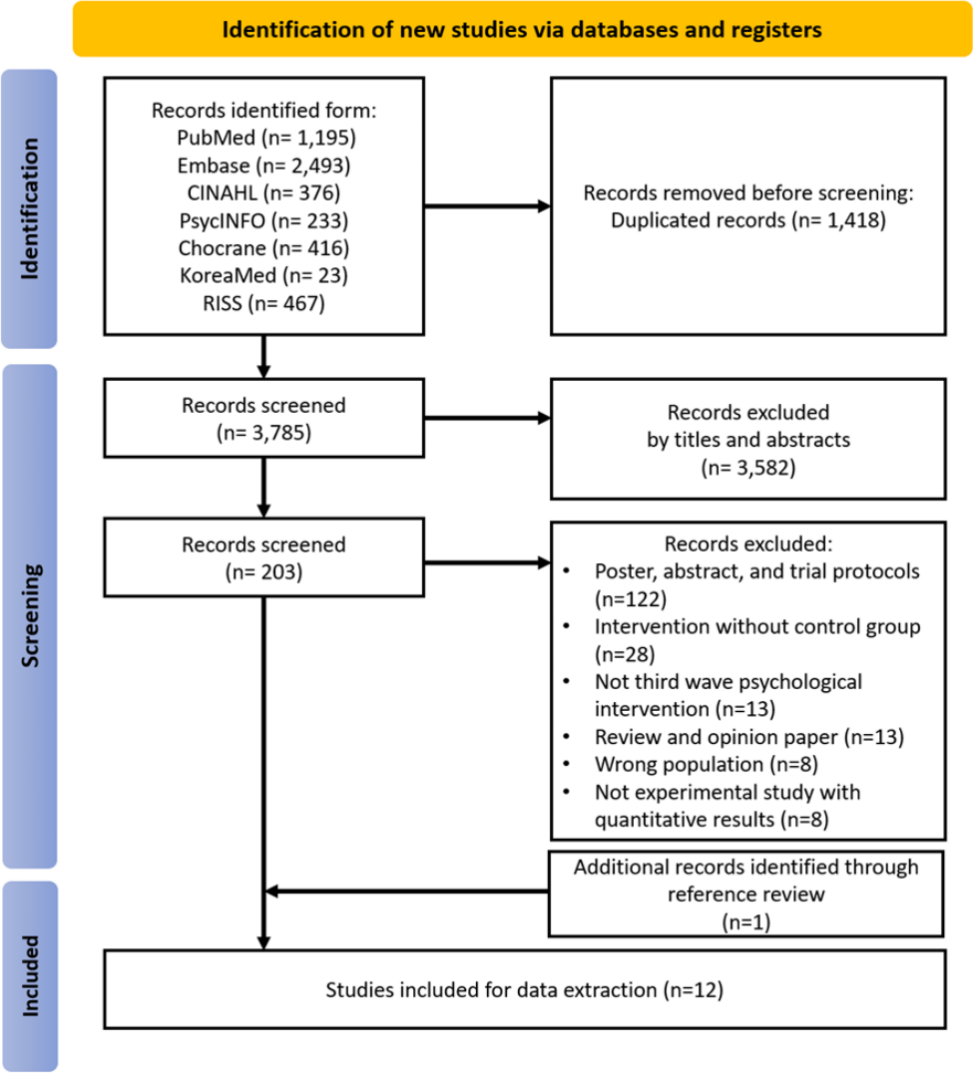
PRISMA-ScR flow diagram

Among the 5,203 available studies, 1,418 duplicates were excluded. The titles and abstracts of 3,785 studies were screened, and 203 studies were initially selected. After the eligibility assessment, 11 studies met the criteria, and we identified one additional record through reference review. Finally, 12 studies were included in this scoping review (Fig. 1).

Studies on cancer caregivers with third-wave CBT have been steadily increasing since 2016, except for 2018, when no published study was identified. The studies were conducted in the United States (n = 8), the Netherlands (n = 2), China, and Taiwan (n = 1, each).

All 12 studies were randomized controlled trials (RCTs). Eight studies were pilot studies [19–26]. Only a single study calculated and secured the sample size for appropriate statistical power [27]. Most studies were designed as 2-arm studies, including single intervention and control groups (n = 10). There were two 3-arm studies; Milbury et al. had two control groups [21], and Köhle et al. had two intervention groups [27] (Table 1).

**Table 1:** Characteristics of the third-wave CBT intervention examining caregivers of cancer patients

Author (year)	Country	Caregivers	Dyad	Patients		Intervention				Control
				**Cancer type**	**Treatment/** **Stage**	**Intervention details**	**Delivery methods**	**Provider**	**Duration**	
**Mindfulness based stress reduction**										
Schellekens (2017) [31]	Netherlands	Partner, relative or friend	No	Lung	Mixed	Mindfulness exercises including body scanning, gentle yoga, and walking meditation	Face to face	MBSR teacher	150 min *8 sessions /8 weeks	Usual care
Kubo (2019) [25]	United States	Primary, informal caregiver	Yes	Mixed	Receiving treatment	Self-paced program providing guided mindfulness meditation tools	Mobile application/web page	N/A	10 to 20 min daily/8 weeks	Attention control
Kubo (2020) [20]	United States	Informal caregiver	Yes	Mixed	Advanced stage	Self-paced program providing guided mindfulness meditation tools OR online virtual class on mindfulness	Mobile application/web page	N/A	10 to 20 min daily OR 120 min weekly/6 weeks	Attention control
**MBSR-based intervention**										
Hsiao (2016) [28]	Taiwan	Spouse	Yes	Breast	Completed active treatments	Body–mind–spirit therapy and assistance coping with stress and marital relationships	Group session	Trained researcher	120 min *8 sessions /8 weeks	Support program for cancer survivors
Price-Blackshear (2020) [29]	United States	Partner	Yes	Breast	Stages 0–3, 1 to 6 years post-diagnosis	Mindfulness-based relationship enhancement program	Prerecorded video	MBSR teacher	60 min *8 sessions /8 weeks	Watching 1 intervention program resource
**Acceptance and commitment therapy**										
Mosher (2019) [19]	United States	Roommate or regular visitor of patient	Yes	Lung	Advanced stage	Intervention targeted all processes of the ACT model of behavioral change	Telephone call	Social worker	50 min *6 session /6 weeks	Supportive expressive intervention and health information education
Köhle (2021) [27]	Netherlands	Partner	No	Patients were not included		Intervention arm ①: Self-help intervention based on ACT and self-compassion with personal feedback.	Web page	N/A	60 to 90 min *6 session /6 weeks ^a^	Attention control
						Intervention arm ②: Self-help intervention based on ACT and self-compassion with automatic feedback.				
Mosher (2022) [24]	United States	Family caregiver	Yes	Gastrointestinal	Advanced stage	Intervention targeted all processes of the ACT model of behavioral change	Telephone call	Clinician or psychologist	50 min *6 session/6 weeks	Health information education
**ACT-based intervention**										
Milbury et al. (2020) [21]	United States	Spouse	Yes	Lung	Stage 4, receiving treatment	Meditation program focused on cultivating mindfulness, compassion, gratitude, and value-based living process of ACT	FaceTime	Psychological counselor	60 min *4 session /4 weeks	Control arm ① : Usual intervention care Control arm ②: Supportive expressive intervention
Milbury et al. (2020) [22]	United States	Spouse	Yes	Brain	Mixed	Meditation program focused on cultivating mindfulness, compassion, gratitude, and value-based living process of ACT	FaceTime	Psychological counselor	60 min *4 session /4 weeks	Usual care
**Acceptance-based cognitive behavioral therapy**										
Trevino (2017) [30]	United States	Primary unpaid caregiver	Yes	Mixed	Receiving treatment	Acceptance-focused cognitive-behavioral therapy for coping with cancer	Telephone call	Social worker	45 to 60 min *7 sessions/ 7 weeks	Usual care
**Langerian mindfulness intervention**										
Geng (2019) [26]	China	Family caregiver	No	Patients were not included		Establishes scenarios for participants to rethink their usual perspectives	Not mentioned	Not mentioned	30 min *4 session	Providing one sample form intervention

^a^ Except for two optional sessions over six weeks after intervention

Among the 12 studies investigated, nine studies targeted patient–caregiver dyads [19–22, 24, 25, 28–30]. Schellekens et al. included both patients and caregivers in the study, but the intervention did not target the patient–caregiver dyad [31]. Köhle et al. [27] and Geng et al. [26] conducted interventions focusing only on caregivers.

### Characteristics of participating caregivers

The studies described caregivers as family, relatives, and friends living with or regularly visiting patients (Table 1). Five studies included only spouses or partners [21, 22, 27–29].

Two studies were conducted with caregivers of lung cancer patients in an advanced stage [19, 21], and two studies were conducted with caregivers of breast cancer patients in the follow-up phase [28, 29]. There was one study that included both gastrointestinal cancer patients in advanced stages and their caregivers in the intervention [24], and one study included brain tumor patients at various stages and their caregivers [22]. Some studies recruited cancer caregivers with patients at specific stages of cancer or treatment (e.g., advanced stage; receiving treatment) without considering the types of cancer [20, 25], while others recruited cancer caregivers without considering the patient’s clinical details [26, 27].

### Characteristics of interventions

The characteristics of the interventions are outlined in Table 1. Classification of interventions was carried out as mentioned in each article and its protocol. There were three MBSR interventions and two MBSR-based interventions. MBSR-based interventions consisted of MBSR components and other types of intervention components such as family resilience [28] or relationship enhancement [29]. ACT (n = 3) and ACT-based intervention (n = 2) also were the most frequently applied interventions. ACT-based interventions integrated positive psychology, mindfulness, and loving-kindness mediation with ACT [21, 22]. Acceptance-based CBT used mindfulness and acceptance, and langerian mindfulness used mindfulness without meditation, unlike other interventions did. No studies that applied BA, CBASP, MCT, or DBT were identified.

Starting with acceptance-based CBT delivered by telephone calls in 2017, all third-wave CBT for caregivers were delivered using digital devices, including email, web pages, mobile applications, and voice or video calls. Mobile phones, including mobile apps (n = 2), voice calls (n = 2), and video calls (n = 2), were the most frequently utilized delivery methods. There were online interventions, for example, those using web pages (n = 3) or email with prerecorded videos (n = 1) to deliver the intervention.

Excluding interventions using mobile applications and webpages, MBSR and MBSR-based interventions were conducted by trained professionals (n = 3). ACT-based interventions were conducted by psychological counselors (n = 2). In the study that provided ACT, trained clinicians or psychologists provided the intervention [24]. Other interventions did not specify the qualifications of the providers or did not report who provided the interventions.

Interventions were conducted for 4 to 8 weeks and consisted of weekly sessions ranging from 45 to 150 min. The most common method was a weekly session for a total of 6 weeks [19, 20, 24, 27] or 8 weeks [25, 28, 29] with a length of approximately 60 min per session [21, 22, 27, 29, 30].

Key components related to the third-wave CBT interventions are summarized in Table 2. To avoid ambiguity in the interpretation, the components specified in the study methods or published intervention protocols were extracted. Mindfulness was the most common component (n = 12), followed by acceptance (n = 7) and value-based process (n = 6). The MBSR and MBSR-based interventions shared mindfulness, and most of them (n = 4) utilized only mindfulness. The MBSR-based intervention by Hsiao et al. used acceptance and value-based living processes with mindfulness [28]. ACT comprises multiple components, including cognitive diffusion, committed action, perspective thinking, compassion, mindfulness, value-based process, and acceptance. Two ACT-based interventions shared five components: acceptance, committed action, mindfulness, compassion, and gratitude. Acceptance-based CBT adopted mindfulness and acceptance in traditional CBT. Langerian mindfulness used mindfulness; however, it did not use meditative techniques, as other interventions did.

**Table 2:** Key components related to the third-wave CBT interventions and targeted outcomes

Author (year)	Key concepts															Targeted outcomes
	Acceptance	Cognitive defusion		Committed action		Compassion		Gratitude		Mindfulness		Perspective taking		Value-based living process		
**Mindfulness-based stress reduction**																
Schellekens (2017) [31]										✔						Caregiver burden, compassion, distress, mindfulness, posttraumatic stress symptoms, relationship satisfaction, rumination
Kubo (2019) [25]										✔						Anxiety, depression, distress, fatigue, mindfulness, pain, posttraumatic growth, quality of life, sleep quality
Kubo (2020) [20]										✔						Anxiety, depression, distress, mindfulness, quality of life
**MBSR based therapy**																
Hsiao (2016) [28]	✔									✔				✔		Anxiety, attachment, depression, psychological well-being, quality of life, sleep quality, stress response
Price-Blackshear (2020) [29]										✔						Anxiety, depression, dyadic adjustment, fatigue, interpersonal mindfulness, mindfulness, perceived stress, relationship quality
**Acceptance and commitment therapy**																
Mosher (2019) [19]	✔	✔		✔						✔		✔		✔		Acceptance of illness, anxiety, depression, distress
Köhle (2021) [27]	✔	✔		✔		✔				✔				✔		Caregiver strain, compassion, distress, general health, positive mental health, posttraumatic growth, psychological flexibility, relational communication style, resilience, sense of mastery
Mosher (2022) [24]	✔	✔		✔						✔		✔		✔		Caregiver burden, engagement in daily activities, psychological flexibility, quality of life, value-based living,
**ACT based therapy**																
Milbury et al. (2020) [21]	✔					✔		✔		✔				✔		Cancer-related stress symptoms, depression, spiritual well-being
Milbury et al. (2020) [22]	✔					✔		✔		✔				✔		Compassion, depression, intimacy, mindfulness
**Acceptance based cognitive behavioral therapy**																
Trevino (2017) [30]	✔									✔						Anxiety, depression, quality of life
**Langerian mindfulness**																
Geng (2019) [26]										✔^a^						Caregiving situation, mindfulness

^a^ Mindfulness without meditative

### Targeted outcomes

Forty-four questionnaires measured 37 outcome variables among cancer caregivers, as summarized in Table 3. The PROMIS® was used to measure various health outcomes, including anxiety, depression, fatigue, pain, and sleep quality. The Hospital Anxiety and Depression Scale (HADS) is the most frequently used measure of depression, anxiety, and psychological distress.

**Table 3:** Targeted outcomes of third-wave CBT interventions

Outcomes	Scale
**Caregiver outcomes**	
Acceptance of the illness	PEACE questionnaire
Anxiety	Hospital Anxiety and Depression Scale (HADS)
	PROMIS®-anxiety
	State-Trait Anxiety Inventory (STAI)
Attachment	Experiences in close relationships revision scale (ECR-R)
Caregiver burden	Self-Perceived Pressure due to Informal Care (SPPIC)
	Zarit burden interview
Caregiver strain	Caregiver Strain Index (SCI)
Caregiving situation	Caregiver Reaction Assessment (CRA)
	Positive Aspects of Caregiving Scale (PAC)
Compassion	Self-Compassion Scale (SCS)
	Self-Compassion Scale Short-Form (SCS-SF)
Depression	Beck Depression Inventory (BDI-2)
	Center for Epidemiological Studies Depression Scale (CES-D)
	PROMIS®-depressive symptom
Distress	The National Comprehensive Cancer Network Distress Thermometer
Distress-psychological	Hospital Anxiety and Depression Scale (HADS)
Dyadic adjustment	Dyadic Adjustment Scale (DAS)
Engagement in daily activities	PROMIS®-social roles and activities
Fatigue	Brief Fatigue Inventory
	PROMIS®-fatigue
General health	RAND 36-general health
Healthcare use	5 domains (including outpatients visits, and overnight hospitalization) in past 3 months at baseline and over the study periods
Intimacy	Personal Assessment of Intimacy in Relationships Inventory (PARI)
Mindfulness	Five Facet Mindfulness Questionnaire (FFMQ)
	Five Facet Mindfulness Questionnaire (FFMQ-SF)
	Langer Mindfulness Scale (LMS)
	Mindful Attention Awareness Scale (MAAS)
Mindfulness-interpersonal	Interpersonal Mindfulness Scale (IMS)
Pain	PROMIS®-pain intensity, pain interference
Positive mental health	Mental Health Continuum-Short Form (MHC-SF)
Posttraumatic symptoms	Impact of Events Scale (IES)
Posttraumatic growth	Posttraumatic Growth Inventory (PTGI)
	Posttraumatic Growth Inventory-Short Form (PTGI-SF)
Psychological flexibility	Acceptance and Action Questionnaire II (AAQ-II)
Psychological well-being	Meaning in Life Questionnaires (MLQ)
Quality of life	Caregiver Quality of Life Index-Cancer (CQOLC)
	Short Form 12 health-related quality of life (SF-12 QoL)
	PROMIS®- global health
Relational communication style	Active Engagement Scale
Relationship quality	Quality of marriage index (QMI)
Relationship satisfaction	Investment Model Scale-Satisfaction Subscale (IMS-S)
Resilience	Brief Resilience Scale (BRS)
Rumination	Ruminative Response Scale-Brooding Subscale (RRS-Br)
Sense of mastery	Pearlin Mastery Scale (PMS)
Sleep quality	Medical Outcomes Study Sleep Scale (MOS)
	PROMIS®-Sleep disturbance
Spiritual well-being	Functional Assessment of Cancer Therapy-Spiritual Well-being Scale (Fact-Sp)
Stress-cancer related	Impact of Events Scale (IES)
Stress-perceived	Perceived Stress Scale (PSS)
Stress-response	Salivary cortisol levels
Value-based living	Valuing questionnaire
**Patient’s outcomes**	
Quality of life	Functional Assessment of Cancer Therapy-General (FACT-G)
	European Organization for Research and Treatment of Cancer Quality of Life Questionnaire-Global Quality of Life Subscale (EORTC QLQ-GHS)
Symptoms	Global Symptom Interference Subscale of the MD Anderson Symptom Inventory (MDASI)
	Interference Subscale of the Fatigue Symptom Inventory (FSI)
	MD Anderson Symptom Inventory-Brain Tumor (MDASI-BT)
	PROMIS®-pain interference, pain severity, dyspnea avoidance, fatigue, sleep disturbance

**Fig. 2:**
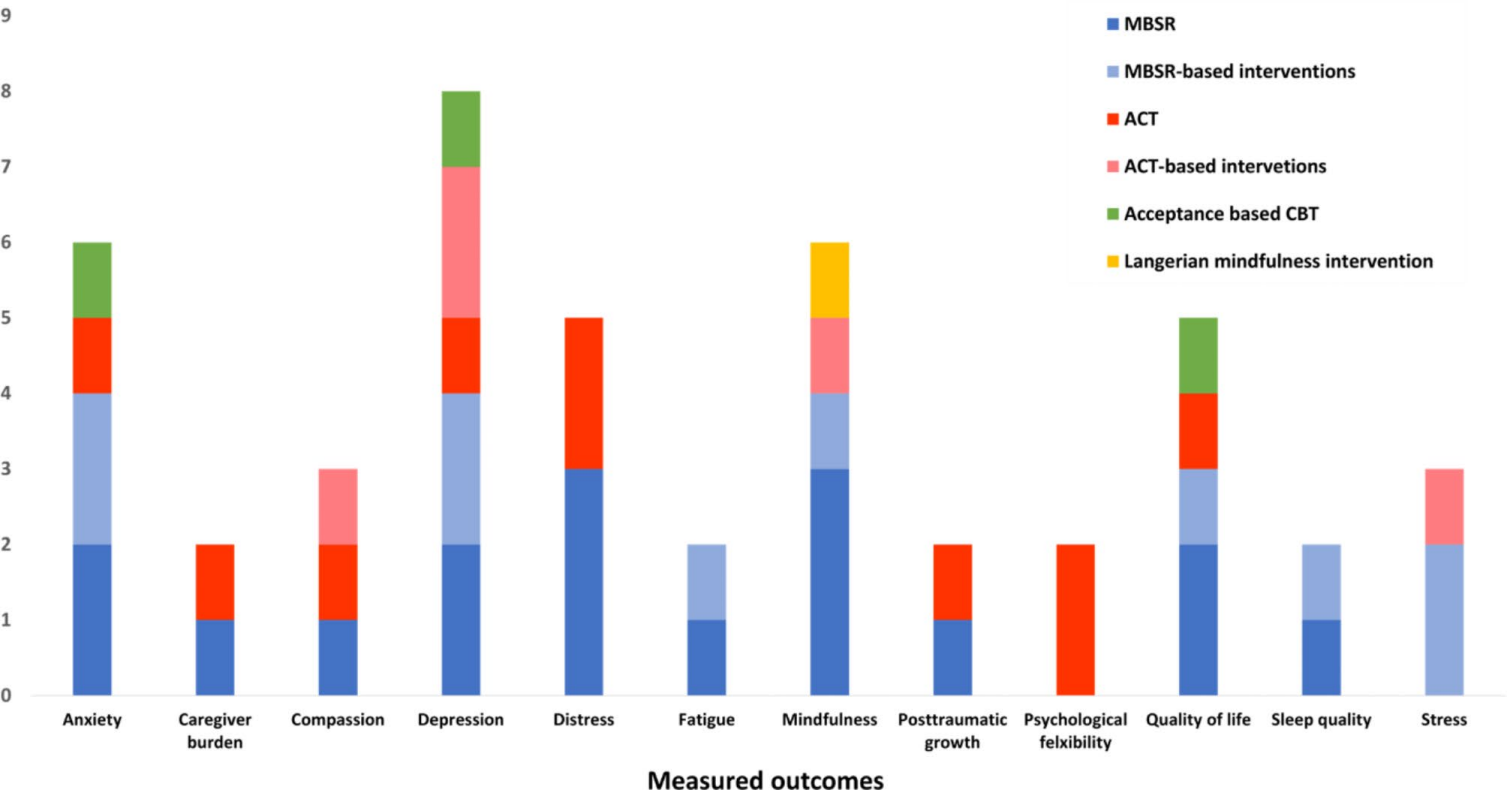
Frequency of caregiver outcomes measured more than once. *Note.* MBSR: mindfulness-based stress reduction; ACT: acceptance and committed therapy; CBT: cognitive behavioral therapy

Frequently identified outcomes of third-wave CBT are summarized as a graph (Fig. 2). Depression was the most frequently measured outcome (n = 8), followed by anxiety (n = 6), mindfulness (n = 6), distress (n = 5), and quality of life (QoL, n = 5).

The primary outcomes of MBSR and MBSR-based interventions were anxiety (n = 4), depression (n = 4), and mindfulness (n = 4). The interventions also evaluated relatively diverse outcomes, including QoL and distress (n = 3, respectively), and were the only interventions aimed at improving fatigue and sleep quality (n = 2, respectively). The main outcomes of ACT and ACT-based intervention were depression (n = 3), distress (n = 2), compassion(n = 2), psychological flexibility (n = 2). ACT and ACT-based interventions are more interested in various psychological outcomes than intervention group of MBSR, which was more symptom-focused. The following outcomes are only addressed in ACT-related interventions; Intimacy, psychological flexibility, resilience, sense of mastery, spiritual well-being, value-based living. The Langerian mindfulness intervention was aimed at improving mindfulness (n = 1). Acceptance-based CBT tried to intervene anxiety (n = 1), depression (n = 1) and improve QoL (n = 1).

## Discussion

We identified five types of third-wave CBT for cancer caregivers investigated over the last 20 years. The most common intervention was MBSR and ACT. MBSR has a flexible structure, takes less time than other interventions and is widely used in nonclinical populations [10]. For this reason, MBSR was the most popular intervention in previous studies on caregivers of elderly individuals [14].

The delivery of third-wave CBT has evolved over time. We noticed a growing trend of intervention using digital devices after 2017. In this review, third-wave CBT has been delivered in various ways, such as voice or video calls, web pages, and mobile applications, moving beyond previous face-to-face delivery methods. This is in contrast to a previous mindfulness intervention study conducted on palliative caregivers in 2016 in which 90% of interventions were provided face-to-face [32]. Interventions using digital devices are accessible at any time and place, making them tremendously advantageous for cancer caregivers, who have a sizable temporal burden [33]. In our review, the participants of two studies using mobile applications positively evaluated the intervention based on its ease of access [20, 25].

In addition to the modes of delivery, the structure of digital interventions needs to be considered. The ineffectiveness of digital health interventions could be derived from the insufficient structure of the intervention when compared to traditional face-to-face treatment [34]. CBT draws out human emotions from unmeasurable areas, observes behavior, and manipulates the configuration of behavior to verify its effectiveness through re-executable experiments; as such, the structure of the treatment is emphasized [5]. Two interventions using mobile applications in our review were less structured programs that did not adhere to essential steps in CBT (i.e., case conceptualization) and were not effective when the outcomes of the intervention and control groups were compared.

One notable disadvantage of interventions using digital devices is that they imply less interaction with the therapist. CBT, which values the relationship with the therapist, still lacks sufficient evidence regarding mobile or web-based interventions that do not require the user to communicate directly with the therapist [33]. Two interventions using the web page and the mobile app without interactions with the therapist failed to demonstrate effectiveness on caregivers [20, 25], except for a positive effect in the domain of mindfulness [25].

Involving the therapist, even indirectly, in the intervention can be one solution in this case. The results of the intervention by Köhle et al. using a webpage indicate that the scores for positive mental health, psychological flexibility, self-compassion, sense of mastery, and relational communication style were higher than those for interventions involving personalized feedback via email [27]. Therefore, it is essential to develop an effective intervention while ensuring ease of use through advanced technology when planning future studies.

In previous systematic reviews of web-based and mobile applications targeting health care workers, digital devices were not yet an adequate substitute for face-to-face interventions [35]. However, digital technology may be more suitable for helping individuals as a complement to face-to-face therapy for managing relatively mild emotional distress [35, 36]. We should continue experimenting with digital technologies and find practical applications for them.

Most of the interventions targeted patient–caregiver dyads. Some patient–caregiver dyad programs reported promising outcomes, and therefore, dyad intervention has been emphasized in the literature [37, 38]. The studies included in our review also displayed some positive results. Hsiao et al. found that during the group session, depression and stress measured by salivary cortisol levels within 45 min after waking up were significantly reduced, and sleep quality, QoL, and mental well-being were improved [28]. Milbury et al. reported a significant group effect of the decrease in depression in patient–caregiver dyads who received couples-based mindfulness meditation intervention [21].

Some interventions indicated improvement in patient health outcomes more clearly than caregiver outcomes. MBSR interventions for patient–caregiver dyads by Schellekens et al. [31] and Kubo et al. [25] found that the QoL of patients measured by the Global Quality of Life subscale of the European Organization for Research and Treatment of Cancer Quality of Life Questionnaire (EORTC QLQ) and Functional Assessment of Cancer Therapy General Scale (FACT-G) was significantly improved, whereas the partners’ QoL measured as distress or burden was not influenced by the interventions. Likewise, dyad ACT-based interventions by Milbury et al. did not improve the psychological health of the caregivers but had a statistically significant positive effect on the patient’s cognitive and cancer-related symptoms [21]. Given that patients’ distress interacts with that of family members [39], improving patient outcomes can ultimately be beneficial to caregivers.

Whether dyad interventions help improve the quality of relationships is debatable. Hsiao et al. reported that immature attachment signaled by, for example, anxiety and avoidance in relationships decreased in relationships between partners [28]; however, Price-Blackshear et al. found that dyadic adjustment and relationship quality worsened after the intervention in the dyad meditation group [29]. A study by Price-Blackshear et al. reported that coordination and relationship quality in individual meditation groups improved over time [29]. These results may indicate that caregivers desire to receive independent support in caregiving settings [32].

Most studies were interested in depression, which was also one of the most frequently measured outcomes in previous reviews about psychosocial intervention [4]. In our review, the interventions of Hsiao et al. [28] and Milbury et al. [21] both demonstrated positive effects of third-wave CBT on caregivers’ depression. The prevalence of depression in cancer caregivers is about 30% [2], which calls for the use of appropriate intervention approaches. Mindfulness-based interventions have demonstrated effectiveness in reducing the severity of depression in a wide range of individuals, with or without the disease [40].

All 12 studies were randomized trials, but only one study estimated and secured an appropriate sample size. The results should thus be interpreted with caution, as the quality of the study has not been evaluated, and improvements in methodology are recommended before conclusions can be drawn about the efficacy of third-wave CBT for caregivers of cancer patients. Moreover, there are not enough data on the long-term outcomes of interventions.

There are some limitations of this review. We included only experimental studies that quantitatively confirmed the results of the interventions. In addition, the search period was limited to the last 20 years based on the start of active clinical application of third-wave CBT, and data from the 1980s to 2000, when the third-wave CBT was first introduced, were not included.

## Conclusions

Over the past 20 years, studies targeting caregivers of cancer patients with third-wave CBT have increased. Most of the interventions have been dyadic, using mindfulness with meditation, and the delivery methods have continuously replaced with digital techniques. Depression has been the most frequently targeted outcome of third-wave CBT among those caring for cancer patients. For further evidence to support the application of third-wave CBT for cancer caregivers, further well-designed studies need to be conducted, and the results of randomized controlled trials need to be synthesized to provide evidence to identify appropriate interventions for caregivers of cancer patients.

### Electronic supplementary material

Below is the link to the electronic supplementary material.

Supplementary Material 1

## Data Availability

All data generated or analyzed during this study are included in this published article.

## Abbreviations

*ACT*: Acceptance and Commitment Therapy
*BA*: Behavioral Activation
*CBASP*: Cognitive Behavioral Analysis System of Psychotherapy
*CBT*: Cognitive Behavioral Therapy
*DBT*: Dialectical Behavioral Therapy
*MBCT*: Mindfulness-Based Cognitive Therapy
*MBSR*: Mindfulness-Based Stress Reduction
*MCT*: Meta-Cognitive Therapy
*QoL*: Quality of Life
