# Supplementary material for: Third-wave cognitive behavioral therapies for caregivers of cancer patients: a scoping review
Source: BMC Complement Med Ther. 2023 Oct 11;23:360. doi: 10.1186/s12906-023-04186-3 (PMC10566119; doi:10.1186/s12906-023-04186-3)
Supplement: Supplementary file 1 — Supplementary Material 1 [file 12906_2023_4186_MOESM1_ESM.docx]

**Additional Files**

**Supplementary Table 1.** Search terms

| Key word | Mesh term (in subject headings) | Search term (in title and abstract) |
| --- | --- | --- |
| Cancer | Neoplasms | neoplasm OR neoplasms OR cancer OR cancers OR tumor OR tumors OR tumor OR tumors OR malignancy OR carcinoma OR adenocarcinoma OR choriocarcinoma OR leukemia OR leukemia OR sarcoma |
| Caregiver | Caregivers | caregiver OR caregivers OR carer OR carers OR "family carer" OR "family carers" OR "family member" OR "family members" OR "informal carer" OR "informal carers" OR relative OR relatives OR spouse OR spouses OR partner OR partners OR kin OR couple OR couples OR dyad OR dyads |
| Third wave cognitive behavioral therapy | Acceptance and Commitment Therapy OR Mindfulness OR Dialectical Behavior Therapy | "third wave" OR "Acceptance and Commitment Therapy" OR ACT OR "acceptance adj*" OR "commitment adj*" OR "Behavioral activation" OR "Behavioral activation" OR "The cognitive behavioral analysis system of psychotherapy" OR CBASP OR "Metacognitive therapy" OR "mindfulness based stress reduction" OR "mindfulness-based stress reduction" OR MBSR OR "mindfulness based cognitive therapy" OR "mindfulness-based cognitive therapy" OR MBCT OR “dialectical behavior therapy” OR “dialectical behavior therapy” OR DBT OR “functional analytic psychotherapy” OR “functional analytic psychotherapy” OR FAP OR “Integrative Behavioral Couple Therapy” OR “Integrative Behavioral Couple Therapy” OR “IBCT” OR meditation* OR "loving-kindness meditation" OR "loving kindness meditation" OR "mindful self-compassion" OR "mindful self-compassion" OR "compassion focused" OR "compassion-focused " OR "compassionate mind" OR "compassion cultivation" OR "cognitively based compassion" OR "cognitively based compassion" OR "body-mind" OR "mind-body" |
| Intervention |  | program OR programs OR intervention OR interventions OR treatment OR treatments OR strategy OR strategies OR technique OR techniques OR training OR trainings OR therapy OR therapies |
